# Perceptions and Experiences of Orthodox Health Practitioners and Hospital Administrators towards Integrating Traditional Medicine into the Ghanaian Health System

**DOI:** 10.3390/ijerph182111200

**Published:** 2021-10-25

**Authors:** Irene G. Ampomah, Bunmi S. Malau-Aduli, Abdul-Aziz Seidu, Aduli E. O. Malau-Aduli, Theophilus I. Emeto

**Affiliations:** 1 College of Public Health, Medical and Veterinary Sciences, James Cook University, Townsville 4811, Australia; irene.ampomah@my.jcu.edu.au (I.G.A.); abdulaziz.seidu@my.jcu.edu.au (A.-A.S.); aduli.malauaduli@jcu.edu.au (A.E.O.M.-A.); 2 Department of Population and Health, University of Cape Coast, Cape Coast P.O. Box UC 182, Ghana; 3 College of Medicine and Dentistry, James Cook University, Townsville 4811, Australia; bunmi.malauaduli@jcu.edu.au; 4 World Health Organization Collaborating Centre for Vector-Borne and Neglected Tropical Diseases, James Cook University, Townsville 4811, Australia

**Keywords:** Ashanti region, hospital administrators, integrated healthcare, orthodox health practitioners, traditional medicine

## Abstract

The government of Ghana has been piloting traditional medicine (TM) integration in 17 health facilities across the country. However, the nature of current practice of integrated healthcare has not been thoroughly explored. This paper sought to explore the experiences and recommendations of orthodox health practitioners and hospital administrators in the Ashanti region regarding the practice of integrated healthcare in Ghana. The study adopted a qualitative, phenomenological approach involving 22 interviews. Purposive sampling technique was used in selecting study participants. Framework analysis was used to draw on the experiences of participants relating to TM integration. Participants were knowledgeable about the existence of integrated health facilities and stated that TM integration has created options in health services. However, participants deemed the integrated system ineffective and attributed the inefficiency to poor processing and certification of TM products, opposition of medical doctors to TM usage, absence of a protocol to guide the integration process, and inadequate publicity. Professional training of TM practitioners and inclusion of TM in medical school curriculum could improve collaboration between the health practitioners. Future research should focus on assessing the opinions and involvements of TM practitioners regarding the integration of traditional therapies into national health systems.

## 1. Introduction

Traditional medicine (TM) comprises ways of treating, preserving, and/or promoting health before the existence of orthodox healthcare [1]. The World Health Organization defines TM as the sum total of knowledge and practices, whether explicable or not, used in the diagnosis, prevention, and elimination of physical, mental, and social imbalance, relying exclusively on practical experience and observation handed down from generation to generation, whether verbally or in writing [2]. TM includes a range of health practices that incorporate plants, animals, vegetables (mineral-based medicines), spiritual/faith healing, and exercise applied solely or in combination to promote or maintain wellbeing as well as treat, diagnose, or prevent ailments [3]. In Ghana, TM comprises the use of medicinal plants and faith/spiritual healing [4,5]. However, in this study, TM refers to products such as medicinal plants, barks, and roots, whether processed or not, that are used for curative purposes. 

Global communities have used TM as part of primary healthcare [6]. Statistics have shown that about 80% of the African population rely on TM as the first line of healthcare [3,7]. The utilisation of TM in low/middle-income countries including Ghana has increased over the years [8]. However, only a negligible proportion of health service users disclose their use of TM to orthodox health practitioners [9,10,11]. Similarly, the majority of orthodox health practitioners are minimally equipped to appropriately guide service users on TM use [12,13,14]. 

Patients’ demands, coupled with political influence, have contributed to the current interest in merging traditional and orthodox health systems into what is now labelled an integrated health system [15]. For example, in most Asian countries, the use of TM is prevalent, although the orthodox health system is readily available. In China, TM accounts for about 40% of all healthcare services delivered and is used to treat about 200 million service users yearly, whereas in Japan, 60–70% of orthodox medical doctors prescribe TM for service users [3]. For developing countries such as Zambia, Nigeria, Mali, and Ghana, TM is used particularly among rural residents in treating children with fever at home because it is readily available [3]. The inclusion of complementary and traditional medicine (TM/CAM) in medical schools’ curricula in the United Kingdom and United States of America has also been highlighted in the literature [3]. Clearly, TM is increasingly being adopted into the orthodox health system as well as the medical curriculum [15,16]. This implies that the orthodox health system (knowledge, practices, organisation, and social roles of medicine in westernised cultures [17]) has moved from an opposing viewpoint to a gradual acceptance of integration with TM [15,18]. The practice of integrated health system engages the orthodox and traditional health systems in delivering health service to populations. TM integration into formal health systems is categorised into three groups (integrative, inclusive, and tolerant health systems) on the basis of the level of incorporation, mainly in the areas of regulation, education, monitoring, and health financing schemes [3]. 

A country is said to be practicing a tolerant health system if the national health system is solely based on the orthodox health system; however, certain aspects of TM are tolerated [3,19]. In contrast, in integrative health systems, TM is officially accepted and utilised in all aspects of healthcare delivery [3,19]. Integrative health systems are noted for ongoing research, training, and education geared towards TM practice as well as proper inter-professional partnership between orthodox and TM systems [20,21]. Some Asian countries such as Korea, China, Singapore, and Vietnam have effectively incorporated full integration of TM into their formal health schemes [19,21,22,23]. In the aforementioned countries, service users have the option of accessing either traditional or orthodox health therapies or a combination of the two healthcare services in formalised settings. In these settings, TM therapies such as Chinese TM, acupuncture [3], and services such as diagnosis and prescription of TM medications are not only available in both government and privately owned health facilities but are also included in the national health scheme [19]. Conversely, in other countries, an inclusive health system approach is utilised. Here, TM practice is officially accepted but not completely included in national health schemes, and formal training on TM at the tertiary educational level might not be available [19]. For example, some high-income (Australia, Canada, United Kingdom) and low/middle income (Ghana, Mali, Nigeria) countries ascribe to the inclusive health system [3,19]. An inclusive health system is characterised by prevalent challenges such as minimal regulation, education/training, and research in TM practices [3,19], particularly in low/middle-income countries. For example, an inclusive health system as practiced in Ghana means that TM practitioners are recognised as healthcare providers and TM products/medications are available in selected government hospitals [19,24]. Therefore, both the traditional and orthodox health practitioners are expected to collaborate and work within an environment of mutual trust and respect [19]. However, evidence depicts that the Ghanaian integrated health system is not as functional as expected [14]. 

The nature of the Ghanaian health system is such that the orthodox system is well established and supported, while the traditional health system, although widely patronised, is less supported [25]. The orthodox health system receives government or political support and funding and serves as the mainstream health system with developed infrastructures and human resources [26]. TM was introduced into the orthodox health system through a series of health interventions to take advantage of the strengths of both systems. These interventions include the formulation of a TM policy in 2005, establishment of the TM council in 2010 to oversee the activities of TM practitioners, inauguration of TM into the tertiary educational system, and establishment of the Centre for Scientific Research into Plant Medicine in 1975 [5,24,27]. Since 2012, TM units have been created in 17 health facilities across the country [28]. All these attempts were made to standardise TM practice and promote its incorporation into the formal health system. However, the Ghanaian health system is non-functional due to weak cross-referrals within the system [5], inadequate knowledge about the practice of integrated health among service users, absence of written documents about integration [25], and the poor attitude of orthodox health practitioners towards TM usage [29].

Some orthodox health practitioners in Ghana view the traditional health system as unscientific and dishonest; therefore, they refrain from referring service users to its practitioners [29]. It is reported that orthodox health practitioners often intimidate service users when they patronise TM before utilising orthodox health services [5]. Evidence from the literature has also indicated that health service users in Ghana access both TM and orthodox treatments without informing orthodox health practitioners [24]. The clinical effect of such client-initiated integration might be unsafe [15,30]. Therefore, the practice of an integrated health system without strong contributions from orthodox health practitioners could result in poor delivery of health services, which could negatively affect the health of the population. Furthermore, a study has highlighted that the government of Ghana recognises the operations of only few TM-oriented institutions such as the Centre for Scientific into Plant Medicine (CSPM) and the TM department at Kwame Nkrumah University of Science and Technology (KNUST) [5]. The activities of many TM practitioners are not recognised and/or regulated. Hence, the traditional health system is poorly governed. Based on the above discussions, the inclusive health system in Ghana is clearly not effective. The ineffectiveness of the health system might render the government’s efforts to achieve an integrative system futile. An effective TM integration into the Ghanaian health system would not only boost the development of the traditional health system but also broaden the scope of health delivery in Ghana. To achieve this, a holistic approach to evaluating the nature of integration is required, including the exploration of the experiences and recommendations of orthodox health practitioners and hospital administrators regarding TM integration into the Ghanaian health system. 

It has been reported that integration is achieved at three levels: the individual (patient/client, health practitioners), institutional (health facilities), and societal (professional/regulatory level/health policy/system) levels. Integration at the individual level comprises the interaction between and among service users, orthodox medicine (OM) practitioners, and TM practitioners, while institutional integration is achieved at the health facilities level where hospital administrators also operate. Societal integration focuses on political will and health policy frameworks [5,15,31,32]. The acceptance of TM integration by orthodox health practitioners and hospital administrators, regardless of their place of operation, is critical to the success of the integration because they are key stakeholders in the health delivery system [6]. A successful TM integration in Ghana at the local and national level would demand active collaboration between all stakeholders [21]. Ghanaians should be enlightened on the official integration of TM into the health system as well as monitor the operations of TM practitioners and products to ensure quality of healthcare. 

Earlier integrated health studies conducted in Ghana [25,26] targeted orthodox health practitioners within urban settings (Accra and Kumasi metropolis), excluding those who practice in rural and remote areas. This emphasises the needs for more inclusive studies that explore the experiences of orthodox health practitioners in both urban and rural settings. Furthermore, previous integrated health research conducted in Ghana focused mainly on the interaction between the two health practitioners (TM and OM) and service users [5,21,25,33]. In this study, the scope of integrative health research is extended to include the experiences and recommendations of hospital administrators since their managerial roles such as disseminating information, supporting change by encouraging employees to partake in implementing a health intervention and distribution of resources within health facilities [34] could influence the success of TM integration.

The merits associated with the practice of an integrated health system could only be achieved when stakeholders in the health system including, orthodox health practitioners and hospital administrators, share common and coherent goals towards the practice of integrated healthcare [15]. Therefore, this study explored the perceptions, experiences, and recommendations of OM health practitioners and hospital administrators regarding TM integration into the Ghanaian health system using Park and Canaway’s conceptual framework for integrating TM into national health systems [6]. The adaption of a conceptual framework in this study could help explain the relationship among the main concepts of integration as well as offer directions on how the Ghanaian integrated system might be enhanced.

### Theoretical Framework

The conceptual framework for integrating TM into national health system was formed to explain the vital role TM plays in various health systems, which is crucial to the achievement of universal health coverage [6]. The framework was adapted for the study because it takes into account the broader elements that affect TM integration. Such elements include population/contextual characteristics, consumer experience, health architecture, health governance, and financing [6]. The framework explains that population/contextual characteristics of a country including population structure, geographical setting, socio-cultural beliefs, historic use of TM, and perceptions about care can either hinder or promote TM integration [6]. For example, when a given population is familiar with TM and trusts its use, then TM integration would be accepted. Consumer experience describes the level of TM integration into the health system. The framework indicates that consumer experience is impacted by health accessibility or capability, the type of health practitioners available, individual preferences, and the fulfilment users derive from the health system [6]. This means that an improved integrated health system connects service users to a team of traditional and orthodox health practitioners. This helps service users to experience integration and stability in accessing healthcare [6,35]. 

**Figure 1:**
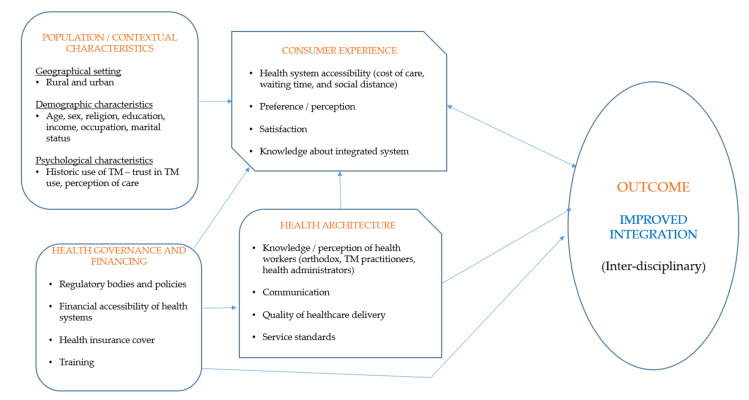
Conceptual framework for integrating TM into health systems. Source: Adapted from Park and Canaway [6].

Health governance and financing element of the framework emphasises how political administration shapes health systems through policies and financing. For example, the inclusion of TM into national health cover as well as the enactment of TM policies could positively influence integration. Finally, health architecture explains the quality of healthcare delivery in a country. This includes the influence and involvement of healthcare practitioners in the integration process. The involvement of health practitioners reflects in their perceptions and knowledge about integration, exposure to TM practice, and communication within the health system (Figure 1) [6]. The framework has been previous employed to study the practice of integrated health in Asia and the Western Pacific [6]. However, to the best of our understanding, it has not been used to investigate integrated health systems in sub-Saharan Africa, particularly Ghana.

This study is a component of a larger research that evaluated the enablers and barriers to TM integration into the formal health system of Ghana by investigating the perceptions, experiences, and recommendations of community members, health practitioners, and hospital administrators in the Ashanti region. This study focuses on two components of the framework: (1) Health governance and financing (2) Health architecture. 

## 2. Materials and Methods

### 2.1. Ethics Approval

Ethics approval for the study was obtained from the James Cook University Human Ethics Committee, Australia (H8239), and the Ghana Health service Ethics Committee, Ministry of Health, Ghana (GHS-ERC003/05/20). All methods and procedures involved in the study were conducted in accordance with the Declaration of Helsinki on ethical principles in conducting human research.

### 2.2. Study Design

This study adopted a qualitative method, specifically, a phenomenology qualitative design which allowed the participants to freely express their lived experiences [36] pertaining to the practice of integrated healthcare in Ghana. The adoption of a qualitative approach facilitated maximal interaction between the researchers and the participants, leading to the emergence of a meaningful collaborative outcome [37,38]. The phenomenology design was suitable for the study because it helped in establishing a combined description of the nature of experiences of the study participants relating to the phenomenon under study. This description comprises of ‘what’ participants experienced and ‘how’ they went through it [36].

### 2.3. Study Setting

**Figure 2:**
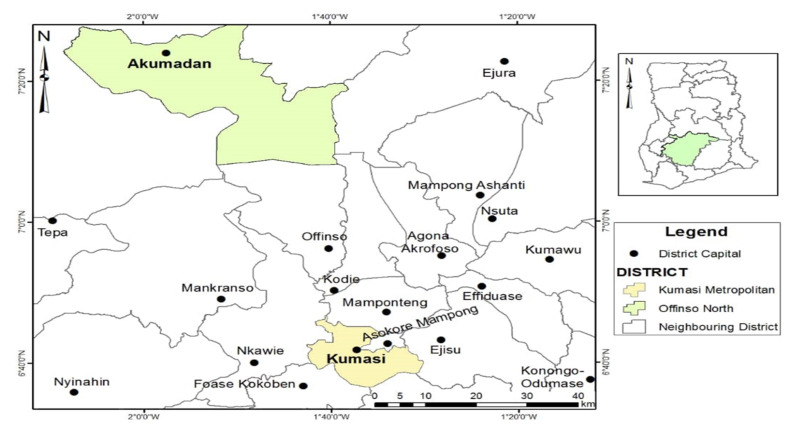
Map of Ashanti region showing the study sites (Kumasi metropolis and Offinso north district). Source: GIS unit of the Department of Geography and Regional Planning [41].

The study setting was the Ashanti region of Ghana. The region is located in the middle belt of Ghana within latitudes 5°50′ N and 7°30′ N, longitudes 0°15′ W and 2°20′ W [39]. The region was chosen because it is the most populous region in Ghana encompassing every socio-economic, ethnic, and cultural backgrounds in the country, accounting for 19.4% of the total population [39,40]. The Kumasi metropolis is the capital of the Ashanti region with three integrated health facilities and a TM training department at the KNUST [5,24], while Offinso north district represents the district with the least population size in the region, with no integrated health facilities. The Kumasi metropolis and Offinso north district were selected as exact study sites with the intention of establishing rural–urban differences or similarities relating to the perceptions and experiences of study participants to the practice of integrated healthcare in Ghana (see Figure 2).

### 2.4. Target Population and Recruitment Strategy

The study targeted orthodox health practitioners (medical doctors (MD), pharmacists (PM), nurses (NS) and hospital administrators (HA) in the Kumasi metropolis and Offinso north district. These groups were targeted because they are stakeholders in the Ghanaian health system. Hospital administrators are responsible for implementing government policies/initiatives such as dissemination of health administration information, distribution of resources, and encouraging employees to participate in the execution of health programmes at the health facility level [34], while orthodox health practitioners also deliver healthcare services to users. The roles these stakeholders play in the health system make them eligible to provide responses relating to health governance, financing, and health architecture components of the framework. Hence, their perceptions and experiences would truly reflect the nature of integrated health practiced in Ghana. Participants were recruited using purposive sampling technique. Purposive sampling used in the recruitment process offered the flexibility required to select eligible participants and maximise differences in the sample [25].

### 2.5. Data Collection Period

The data collection period spanned from February to May 2021. Three research assistants (two males, one female) were trained in a 5 h workshop and provided with interview guides to help with data collection. The training helped the research assistants to understand the objective of the study and aided their adherence to the interview protocol. 

### 2.6. Data Collection Procedure

Data were collected using face-to-face in-depth individual interview procedure. This method was used because it offered the flexibility and privacy for the participants to air their views. The interviews were conducted in both English and Twi (the dominant local dialect in the study setting) at conducive places devoid of third-party intrusions. All the interviews were conducted in the offices of the study participants. The interviews lasted between 45 to 90 min, and all interviews were audio recorded.

Both verbal and written informed consent were sought from participants before the commencement of the interviews. Since TM use is prevalent in the study area, the issue of bias was neutralised by using a training manual to train and guide research assistants as to how the study questions should be asked. This helped in avoiding introduction of interviewers’ personal preferences as far as TM integration is concerned. Additionally, the first author (I.G.A.) was present at the first five interviews to ensure exactitude and uniformity in the interview process. However, no interaction transpired between IGA and the participants. 

The research assistants are conversant with qualitative research approaches. Therefore, the interviews were conducted according to acceptable standards in qualitative research. Assistants were stationed at the study area at the time of the research. Research assistants also prepared field notes by noting their observations and interactions with participants. 

At the beginning of the interview, participants were asked to provide demographic details (sex, age, years of practice, and specialty) about themselves. Semi-structured interview questions were used to explore participants’ views about TM integration into the formal health system. Interview questions were developed based on the framework for integrating TM into national health systems and included questions such as perceptions about the health systems, knowledge about TM integration, communication within the health system, merits of integration, barriers to integration, and measures to promote proper integration among others. Prompts and probes were developed concerning the interview topics, when necessary, to kindle further responses from the participants. Data saturation was reached at the 20th interview, after which two more interviews were conducted, totalling 22 interviews. Saturation occurred at the 20th interview because the additional data gathered did not have unique properties to form a new category [42]. Numbers were assigned to participants to aid anonymity. The data collection instrument was pilot tested before actual data collection commenced. Pilot testing was conducted to ensure that the interview questions were well defined and delivered in a consistent way to avert ambiguity. Reiteration of the interview sessions was not required; however, elucidations were obtained from some of the participants after the data collection period.

### 2.7. Data Analysis

A professional transcriber transcribed all the 22 interviews, and I.G.A. read the transcripts thoroughly. The transcribed data were analysed using Nvivo version 12 software, and framework analysis was the analytic approach employed to identify the perceptions and experiences of the participants in relation to TM integration into the Ghanaian health system. This approach involves five stages of analysis where both inductive and deductive methods were applied. The steps involved in the framework analysis are familiarisation, identification of thematic frameworks, indexing, charting and mapping, and interpretation [28,43]. With the familiarisation stage, I.G.A. read the transcripts and made notes on the major issues. Thematic frameworks (discovery of key concepts in the data) were developed based on the notes made during the familiarisation stage, where the main ideas expressed by study participants were discovered inductively. Indexing was the third stage, where portions of the data were marked as belonging to specific themes or concepts. This stage was also conducted using the inductive approach; hence, the themes emerged freely. The portions of the marked data were arranged in charts corresponding to the identified themes. Finally, mapping and interpretation were conducted by arranging the charted information to illustrate the participants’ knowledge about TM integration as well as their experiences regarding the practice of integrated healthcare in Ghana. At the mapping and interpretation stage, a deductive approach was used by grouping the themes under the main tenets of the framework for integrating TM into national health systems. Preliminary coding and generation of themes were conducted independently by I.G.A. and B.S.M.A. to promote trustworthiness of the results. Crosschecked data had a 90% degree of uniformity, and differences were resolved through deliberations and mutual agreement at a consensus meeting. Three authors (A.A.S., A.E.O.M.A., and T.I.E.) reviewed the themes and quotations to increase the trustworthiness or credibility of the results. Themes were presented along with representative quotes affixed with study participants’ characteristics (for example, Participant 1, MD, Offinso north). The COREQ checklist [44] for reporting qualitative studies was employed in this study (see Supplementary Table S1, COREQ Checklist).

## 3. Results

### 3.1. Characteristics of Participants

There were 22 participants in the study, 16 of whom were orthodox health practitioners (7 medical doctors, 7 pharmacists, 2 nurses) and 6 were hospital administrators. Twelve participants were from the Kumasi metropolis, while 10 were from the Offinso north district, and most (17) of the participants were males. The ages of the participants were between 24 and 49 years. Thirteen of the participants belonged to the Akan ethnic group, while the rest were from the Mole-Dagbani (7) and Ga-Adangbe (2) ethnic groups. In terms of years of experience, this ranged from 1 year to 19 years of practice.

### 3.2. Themes

Nine themes emerged from the data. These themes were mapped under the two components of the conceptual framework. Health governance and financing (regulatory bodies and policies, financial accessibility of health systems, national health insurance cover and training) and health architecture (knowledge about TM integration, perceptions about TM and orthodox health system, communication, quality of healthcare delivery, service standards).

#### 3.2.1. Regulatory Bodies and Policies

Regardless of professional background, participants were aware of the existence of a TM policy/Act, the Food and Drug Authority (FDA) as a regulatory body, and performance of the regulatory bodies. Participants stated that a law had been passed that led to the creation of the TM Act, which spells out how TM should be practiced in Ghana. They also understood the role of the FDA and indicated that the FDA is a body, which supports and regulates TM practice in Ghana because it is tasked with evaluating and certifying the safety of TM products before the products are released into the Ghanaian market for sale. However, the participants were not pleased with the manner in which the regulatory bodies executed their duties. Most of the participants felt that the regulatory bodies were performing abysmally and based their assessment on the presence of uncertified or unlicensed TM products in the health system as well as poor monitoring and evaluation of activities of private TM practitioners.

*“I know there is a law that has set up the TM Act; it captures all issues relating to TM practice in Ghana”.*

*[Participant 4, PM, Offinso north]*

*“…after production, the FDA will come and check the procedures used and once the practitioner is done, they will test the safety of the medicine before they accept it and release it onto the market for sale”.*

*[Participant 1, PM, Offinso north]*

*“From where I sit, I do not think they are doing much. As a facility, the regional health team comes to do monitoring and evaluation. When you look at those in the private practice, I think most of them do not have the license to practice and I am not aware of the council going to monitor their practice”.*

*[Participant 2, HA, Kumasi]*

#### 3.2.2. Financial Accessibility of Health Systems

The participants voiced that the informal delivery of traditional health products, particularly by community-based practitioners, tend to be inexpensive. However, they explained that TM products offered within official or recognised settings, such as licensed chemical/pharmacy shops and clinics, are expensive. An example was cited that malaria treatments offered at formalised TM healthcare settings were costlier than orthodox malaria medications. Hospital administrators also clarified that the high prices of TM products is a key challenge hindering TM integration in Ghana. They explained that clients who patronised TM at formal health settings such as TM clinics (both private and integrated facilities) pay out of pocket, which to them is a barrier to the full patronage of integrated healthcare services because most of the service users are economically challenged.

*“Those TM that are in pharmacies and licensed chemical shops or clinics are expensive. TM treatments for malaria from these avenues are more costly compared to orthodox malaria treatment”.*

*[Participant 1, NS, Offinso north]*

*“Comparatively, the TM clinic that my relative attends; it appears that the cost is higher compared to the orthodox clinics. The medications that she received there for two weeks cost about GHC 1200 which is on the high side if you compared to the orthodox medicine where the medications given were not expensive, not even up to GHC 300”.*

*[Participant 2, HA, Kumasi]*

*“When people come for TM services/products, except for the folder, they pay for everything including the TM products. These products are expensive. So clients paying out of pocket is challenging because most of these people come here without holding even GHC 10 or GHC 20. They (clients) are unable to afford the TM at the TM unit hence accounting for low patronage”.*

*[Participant 6, HA, Kumasi]*

#### 3.2.3. Health Insurance Cover

Study participants considered health insurance as a key strategy in financing health policies. Participants irrespective of their profession or place of operation admitted that the National Health Insurance Scheme (NHIS) is high in the Ashanti region, with most subscribers being poor or economically disadvantaged. Although the NHIS is high, participants stressed that it covers only the mainstream or orthodox health system and contributions from the scheme form a considerable proportion of health facilities’ internally generated funds (IGF). Since premiums from the health insurance contribute greatly to the IGF of health facilities, the inclusion of TM products in the NHIS could serve dual purpose by positively influencing the finances of public health facilities as well as aid health accessibility among users, particularly the poor.

*“The health insurance coverage is high, a lot of people are having the cards but the insurance covers only the cost of orthodox treatments and drugs”.*

*[Participant 5, MD, Offinso north]*

*“It is very high. In terms of percentage, it will be like 70% because when you come in here to our hospital, about 90% of our IGF is from the health insurance. The insurance includes only the mainstream health services”.*

*[Participant 6, HA, Kumasi]*

#### 3.2.4. Training

Participants deemed professional training of TM practitioners an important issue in the integration discourse. They were concerned that TM practitioners did not have formal education and professional training and stated that most TM practitioners acquired knowledge on the practice through informal means as they learnt the profession from their fathers or grandfathers. This according to participants has contributed to orthodox medical doctors not regarding the TM practice as authentic. All the participants believed that medical doctors’ opposition to the traditional health system stemmed from their unfamiliarity with traditional health therapies, making it difficult for them to work with TM practitioners. The medical doctors explained that their non-exposure to TM is creating a barrier to effective integration. They indicated their understanding of how orthodox malarial medications work but felt the same could not be said of the traditional means of treating malaria.

*“Some TM practitioners are not well educated. They will say they inherited the skill from their fathers or grandfathers. Their level of formal training is not too encouraging. So why wont the doctors look down on TM practice”.*

*[Participant 1, NS, Offinso north]*

*“When it comes to the TM, the training is low, people are calling themselves doctors and pharmacists but you do not know where they acquired their knowledge. They just spend some time with their grandparents and they call themselves doctors. So, there is no certificate!”.*

*[Participant 1, HA, Kumasi]*

*“I know how an anti-malarial works but I do not know how the traditional anti-malarial works. I have never been exposed to it but you want me to accept it; I will never accept it and work with them. That is the barrier we are having”.*

*[Participant 3, MD, Offinso north]*

#### 3.2.5. Knowledge about TM Integration

This theme addressed the knowledge level of study participants. Generally, most of the participants, regardless of profession or places of operation, were familiar with some of the interventions implemented to promote TM integration into the formal health system. Participants demonstrated their familiarity with the creation of a TM Department at the Kwame Nkrumah University of Science and Technology (KNUST) and the presence of integrated health facilities in the region. For example, hospital administrators felt that the creation of the TM Department at KNUST serves as a means of training people to be experts in traditional therapies. Similarly, medical doctors and pharmacists explained that the TM Department is under the Faculty of Pharmacy and conducts research into TM with the sole purpose of making the traditional health system an apposite one.

*“There is a TM department at KNUST and it is a four-year program. I know the program is still going on. It is under the Faculty of Pharmacy”.*

*[Participant 4, PM, Offinso north]*

*“At KNUST, there is a department for TM. They train people to become experts in the TM area”.*

*[Participant 5, HA, Offinso north]*

*“At KNUST, there is a department for TM. They are into researching TM and how it can serve as an appropriate healthcare. I know they are under the Faculty of Pharmacy”.*

*[Participant 2, MD, Kumasi]*

Most integrated health facilities in the Ashanti region are located in the Kumasi metropolis, yet participants, irrespective of location of their affiliated health facilities, knew about the existence of such facilities in the region. They believed that the creation of TM departments at public health facilities was an initiative from the Ghana Health Service. Participants attributed their sources of information to both formal and informal channels. As pharmacists received information through formal means such as the circulation of a memo stating the creation of TM unit in the facility, medical doctors and nurses learnt about integration through informal interaction with colleagues. However, hospital administrators became aware of integrated facilities because they work in such facilities. Study participants affirmed that the Kumasi South, Tafo government, and Suntreso government hospitals are the public health facilities with TM departments in the metropolis. They explained that the TM departments are part of the hospitals with a separate reporting system. However, all activities at the department are managed and supervised by the facility’s medical director.

*“Tafo government hospital and I think Kumasi South hospital have this system. I mean the integrated system. I think it is something that the Ghana Health Service is trying to bring on board. I got to know about the integration through my interactions with colleagues”.*

*[Participant 7, MD, Kumasi]*

*“I know of three facilities that operate this integrated system: Suntreso government hospital, Tafo government hospital and Kumasi South hospital. They sent a circular or memo around that from this day; they are bringing TM practitioners so that they would practice in the hospital/facility”*

*[Participant 3, PM, Kumasi]*

*“I know that they have started in some hospitals but not all health facilities. I think the integrated facilities in the region are three. I cannot really remember the source of the information; maybe it was through a conversation”*

*[Participant 1, NS, Offinso north]*

*“The TM unit is part of the hospital; it is part of the units in the hospital. They have their reporting system and at the end of the day, everything gets to the medical director. So, as an administrator I know that there is a structured TM clinic in this facility, therefore we are one of the facilities that deliver integrated services in the region. I became familiar with integrated facilities because I happen to work here”.*

*[Participant 1, HA, Kumasi]*

#### 3.2.6. Perceptions about Traditional and Orthodox Health Systems

In discussing their perceptions about the health systems, the participants described TM as the oldest form of healthcare in Ghana. They believed that TM existed before the advent of colonialism and was effective in treating ailments because the users were getting positive results. Some of the participants concluded that most Ghanaians would seek TM first when the need arises because of their familiarity with the traditional health system. This assertion was popular among participants in the rural setting. Hospital administrators on the other hand were of the view that TM are associated with minimal side effects. According to them, orthodox medicines are synthetic or unnatural; however, the human body tends to be more responsive to TM because it elicits a similar response to food that is consumed.

*“In the past, before the coming of the Whites, our ancestors were using these TM and they were getting the results. So TM has been with Ghanaians for a long time. That is the oldest form of care in Ghana”.*

*[Participant 3, MD, Offinso north]*

*“That has been our way of treating illnesses. The typical Ghanaian or African will not seek modern healthcare if they have an issue. They will try what they know first, that is the traditional therapies. When I was a child, I was asked to take the TM called ’Acheampong’. So, it has been part of us for a long time”.*

*[Participant 5, PM, Offinso north]*

*“I can even say that most of them (TM) come with little or no side effects because they are like the foods that we eat. We all know paracetamol has side effects and so do other orthodox medicines. If you do not read the instructions carefully and you take the medicine, then you would feel dizzy. However, when it comes to the TM, although some of them come with little side effects, most of them do not have side effects”.*

*[Participant 1, HA, Kumasi]*

One of the strengths of the orthodox health system as mentioned by study participants is that the system is evidence-based and supported by science or research. Participants elucidated that orthodox related health activities and treatments could be explained scientifically and their practitioners act in line with laid down principles in the practice. However, they felt that the same could not be said of the traditional health system. An example was cited that healthcare activities such as conducting laboratory test are not considered in traditional healthcare delivery.

*“So, the orthodox system comprise of activities that are supported by science. I cannot do whatever I like because I am a medical doctor. My actions should be science-based. But with the traditional way of treating diseases, laboratory tests are not taken into consideration before certain medications are given to the person who is accessing the care”.*

*[Participant 7, MD, Kumasi]*

In addition to the strengths of the two health systems, the participants identified some opportunities for TM integration. They perceived the availability of medical plants and already existing market for TM as opportunities for promoting TM integration into the Ghanaian health system. Medical doctors declared that Ghanaians’ familiarity with the traditional health system could influence the integrated health system because it is a positive reinforcement. Pharmacists and nurses also considered the availability of medicinal plants as an advantage to integration. They explained that the availability of medicinal plants has the potential of sustaining the integration through constant supply of raw materials for the production of TM products.

*“See, TM is already in existent in our system so people already know of it. So, there is ready market, which is somewhat good for the integration because it will not be new to people”.*

*[Participant 5, MD, Offinso north]*

*“The availability of medicinal plants is an opportunity to boost integration. We do not have to export materials to sustain the TM field. Medicinal plants are already there. We making use of such plants and other things in our ecosystem is a great opportunity for integration”.*

*[Participant 4, PM, Offinso north]*

*“These medicinal plants are all around us. Just look at even our health facility here, there are many plants around us. Most of them are medicines including the Nim tree so we cannot run out of raw materials for TM products, which to me could sustain the integrated system”.*

*[Participant 1, NS, Offinso north]*

#### 3.2.7. Communication

Participants were displeased with the nature of communication within the Ghanaian health system. They narrated that service users seldom shared information on TM use with orthodox health practitioners. Medical doctors and pharmacists believed that the reason for the non-disclosure of TM use by service users is to prevent being judged by the practitioners or the perception that orthodox health practitioners frown at TM usage. Contrarily, nurses believed that service users would only disclose their use of TM upon further probing, especially at the time of taking their medical history. The orthodox health practitioners, particularly medical doctors, felt that they do not offer service users enough health education regarding how, where, and when to seek healthcare. This they believe put service users in a situation where they resort to all forms of treatments when the need arises.

*“Patients hardly tell us their use of TM. I do not know whether it is because patients have the notion that, generally health workers at the hospital frown on TM usage”.*

*[Participant 2, MD, Kumasi]*

*“You have to ask the client’s past medical history. If the patient has taken or applied any TM, they will tell you. So, you have to ask them and they will tell you”.*

*[Participant 1, NS, Offinso north]*

*“…sometimes I feel like we the medical doctors are to be blamed in a way. The part that we do not do well is that we do not offer people enough information on what and when to do what and that is what the patients want. So, we sort of put them (patients) in a state where they have to find other means of care”.*

*[Participant 6, MD, Kumasi]*

Most of the participants agreed that the key to delivering appropriate healthcare services to users is through timely referrals. However, orthodox health practitioners admitted that they do not refer service users to TM practitioners. It emerged from the interviews that TM practitioners are usually delayed in referring service users to orthodox health practitioners, and such referrals were unofficial, mostly by word of mouth. They further clarified that the delay in referring service users to the hospitals usually leads to deterioration of the health conditions of the clients.

*“I have not referred any of my patients to seek care from any TM practitioner. I have not done that before. What I have realised is that, TM practitioners do not refer cases on time. They delay until the patient’s condition has deteriorated before they refer and they do that just by word of mouth”.*

*[Participant 5, MD, Offinso north]*

In terms of publicity, hospital administrators recounted that integrated health facilities were publicised through health education programmes using the media, particularly the radio, as a channel for information dissemination. The key messages delivered during such health programmes include the creation of TM unit in public health facilities and education on the entire TM integration process. Even though hospital administrators narrated the means through which TM integration is publicised in the region, participants felt that publicity about TM integration was not adequate, hence deeming it a challenge to the integration process. According to participants, people cannot access a non-existent service. Hospital administrators openly admitted that institutions including those that offer integrated healthcare services have not done enough to publicise the official integration of TM into the Ghanaian health system because of the absence of directional signs for the integration.

*“We buy airtime and go to the radio station to talk about the integration. So we talk about the creation of TM unit in our facility”.*

*[Participant 1, HA, Kumasi]*

*“I only got to know about the integrated system because of the TM department within the hospital. Aside that, I have not heard of the integration of TM from any other place, which I think is a problem. How do you practice something you do not know? How can people also access a service that they do not know it exist?”.*

*[Participant 4, MD, Kumasi]*

*“As for the publicity, I think it is one of the challenges. Most people do not even know that when they come to the facility, they can have access to TM, except for the clients that are already aware. In this hospital and the others, we have not done enough to publicise the integration. I am the administrator and I know that we do not have directional signs. The publicity is not there”.*

*[Participant 4, HA, Kumasi]*

#### 3.2.8. Quality of Healthcare Delivery

Some participants reported that the orthodox health system is founded on laid down scientific principles and procedures such as triaging and medical examinations. They stated that conducting such health activities improve the quality of healthcare delivered to service users, which in most cases leads to the identification of the root causes of illnesses. Other participants also narrated that the procedural nature of the orthodox health system aid in the identification of gaps in the healthcare delivering chain, since decisions regarding the type of healthcare a client needs do not rest on one health practitioner but on a range of other orthodox health practitioners.

*“Before a medical officer will get to you, there is something we do called triaging. We try to assess your medical situation and condition to get to the bottom of things. So, we ask couple of questions, do physical examinations and if necessary conduct laboratory tests to get to the root of ailments. We do all these things with the aim of delivery quality healthcare to the patient”.*

*[Participant 7, MD, Kumasi]*

*“…mostly I call the doctors and tell them that I have realised that they are prescribing something to patients but I think it should be this or that. I am able to do that because of some commonalities in the practice. For example, there was a pregnant woman who came from a facility, different from ours. They had prescribed ‘ergometry’. If a pregnant woman takes that drug, then it is likely to cause an abortion. So, sometimes, it is not that the doctor does not know what he/she is doing but they might have been a slip of hand where the mind is going faster than the hand. So, I went to the facility and told the doctor that with all due respect, I saw ‘ergometry’ but I think it is ‘ergotamine’, and so he should confirm, so that the patient will come back and he did. You see! At the end, the patient received the best of care. We usually get favourable results where both the doctors and patients will come back to say thank you”.*

*[Participant 2, PM, Kumasi]*

Considering the delivery of integrated healthcare service in the region and Ghana as a whole, all participants mentioned that TM integration has led to availability of options in healthcare services. They believed that TM integration into the formal health system has been beneficial because it makes TM services and products available to service users at approved health facilities, hence increasing the number or range of medications at accredited health facilities for people to choose from. Hospital administrators commented that TM integration has led to the production and delivery of safe TM products and services. To administrators, approved TM undergoes strict scientific scrutiny or evaluation at research centres, therefore guaranteeing the safety of such products. They believed that TM integration has paved a way for a gradual disengagement from counterfeit TM products. They further explained that people who patronise TM services at integrated health facilities go through acceptable and standard ways of healthcare delivery. Hence, the availability of TM departments at the hospitals has led to the delivery of safe health services associated with the TM practice. Interestingly, medical doctors also felt that the inclusion of TM in the formal health system is a strategy for the preservation of indigenous medicines.

*“The patients who patronise services in this facility are open to make a choice whether they want the TM or orthodox medicine. So, there are options for them to choose from. Patients have access to alternative healthcare”.*

*[Participant 3, PM, Kumasi]*

*“Before a TM product is accepted within the integrated system, it goes through some tests and research. It passes through the research centre at Mampong to meet all the standards for the FDA to give approval, so gradually we are separating the good TM from the fake ones”.*

*[Participant 1, HA, Kumasi]*

“Because it is at the hospital, it helps to promote the safety of TM services. At the end of the day, we do not just give TM anyhow. It is according to acceptable standards in healthcare. When a patient comes to the hospital, he/she will go for the folder and go through all the normal things/triaging before he/she sees the TM doctor”.

*[Participant 6, HA, Kumasi]*

*“In fact, the integration is a way of maintaining our indigenous medicines. It is serving as a way to come back to our traditions and culture”.*

*[Participant 5, MD, Offinso north]*

Participants emphasised that integration has led to boosting of database on health accessibility. They explained that the practice of integrated healthcare has created an avenue for data gathering on health accessibility, especially on service users that patronise TM. They felt that through triaging at Out Patient Departments (OPDs), data on TM users are collected that help to track and know the category of people who usually use TM products/services.

*“When a client comes here for TM, he/she goes through the normal health checks at the Out Patient Department for them to check the vitals, weight and pressure. That way, we are able to collect information on people who access TM services, which is improving database on health accessibility”.*

*[Participants 6, HA, Kumasi]*

Despite the positive impact of TM integration on health delivery, orthodox health practitioners and hospital administrators felt that the claim of a particular TM treating a host of diseases is problematic. Pharmacists described this action of TM practitioners as being dangerous to the medical field. To medical doctors, the claims made by TM practitioners about their products put them in a state of confusion and reduce their desire to work with them. Similarly, hospital administrators also added that the claim of one TM treating a number of diseases has discredited the TM system and concluded that once trust has diminished, active collaboration between the two health practitioners becomes challenging.

*“Some people will come and they have hypertension and another person coming with erectile dysfunction. Yet, they will present one TM that can treat all these problems. I think about it and I ask myself how this is possible? So I do not trust those claims; hence do not have the desire to work with them (TM practitioners)”*

*[Participant 6, MD, Kumasi]*

*“TM people at times make many claims about TM. They make so many claims that one TM can cure multiple diseases…. You see! These claims are dangerous to the practice of medicine”*

*[Participant 5, PM, Offinso north]*

*“You will see so many TM practitioners around claiming that one medicine can cure almost everything. So, you wonder if that is truly so. So, it affects how other health practitioners trust the TM system, and if trust is diminished, it affects collaboration”.*

*[Participant 4, HA, Kumasi]*

#### 3.2.9. Service Standards

The participants were of the view that TM products were poorly processed and their certifications were not up to the expected standard. As orthodox health practitioners were more concerned about certification and standardisation of TM products, hospital administrators were mainly displeased with the unhygienic conditions in which some TM products were prepared. Medical doctors and pharmacists perceived poor certification or standardisation of TM products as a challenge to integration because they viewed proper certifications as a requirement to making informed decisions on healthcare delivery.

*“The challenge I have with TM is the quantification of their doses, and probably the hygienic state of its preparation. In addition, they (TM practitioners) do not normally state the side effects of the TM; neither do they provide an antidote to overdose. So, in the case of overdose, what do you do? You do not get answers to these questions. So how do you work with people in a field that they do not give you enough information to work with?”.*

*[Participant 1, MD, Offinso north]*

*“The problem I have as a pharmacist is its (TM) standardisation. How do I know that this is the dosage? Sometimes, they will tell you to use a cup to measure and drink, which is not right”.*

*[Participant 4, PM, Offinso north]*

*“Sometimes, the way it is prepared, the environment, the tidiness and all those things make it non-standardised”*

*[Participant 3, HA, Offinso north]*

When deliberating on the conditions needed for effective implementation of the integration, pharmacists felt that there was no document or protocol that described into details the dynamics or scope of the integration. Participants viewed the absence of such a protocol as a flaw in the implementation process, hence making TM integration in Ghana being far from perfect. They reported that the intervention was imaginary and attributed their assertion to the absence of a documented protocol detailing when and how cross referrals should be conducted and/or how TM practitioners should be integrated into the progression and salary structure within the Ghanaian health system. Participants felt that the absence of a written protocol has created laxity in the system where everyone does what he or she perceives to be right. Pharmacists in the urban study setting mainly raised this concern.

*“There is lack of protocol for integration. We know that there is integration but when do I refer patients. We should know that you have to refer a patient when the temperature is above this or that. The TM practitioners should know that they have to refer if their (clients) are having this or that symptoms. How do we know when to refer to a TM practitioner or a medical doctor? The absence of such a protocol makes interaction in the system problematic. Everyone is doing his/her own thing”.*

*[Participant 2, PM, Kumasi]*

*“The issue is that, how do we integrate them into the salary structure and all that? So, fair wages and salaries commission would have to look at the progression for TM practitioners. The absence of a document spelling out all these dynamics make the integration far from perfect. Is it as if institutions are acting per their understanding, which is a big hindrance to integration”.*

*[Participant 4, PM, Kumasi]*

### 3.3. Recommendations Made by Participants to Enhance TM Integration into the Ghanaian Health System

**Figure 3:**
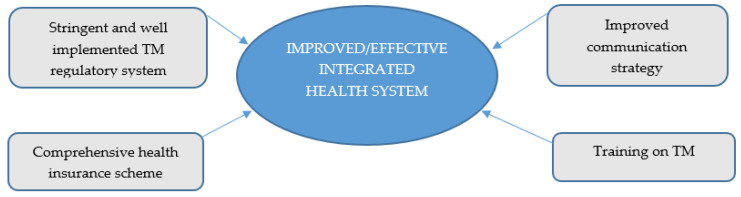
Participants’ recommendations to enhance TM integration into the Ghanaian health system.

Based on the above observations, participants proposed four recommendations to stakeholders including legislations on how to promote effective integration of TM into the Ghanaian formal health system. These recommendations include stringent and well implemented TM regulatory system, comprehensive health insurance scheme, training on TM, and improved communication strategy. Figure 3 sums up the recommendations from study participants.

#### 3.3.1. Stringent and Well Implemented TM Regulatory System

All participants stressed that proper implementation of TM regulatory rules by the appropriate regulatory bodies is required to enhance TM practice and consequently promote its integration into the formal health system. Participants were knowledgeable about the existence of regulatory bodies; however, they were not satisfied with their performance. They proposed that the regulatory bodies, particularly the FDA, could be more decentralised in their operations by establishing sub offices across the country in order to improve monitoring of TM practice and market surveillance.

*“The FDA can have sub associations that can monitor some of these things. I think the law enforcement agencies too can also help. Once a while, the FDA can send people to the pharmacy stores to check if the board has accepted those drugs”.*

*[Participant 2, MD, Kumasi]*

*“I think more stringent measures should be placed on TM preparation. It always comes out that the FDA has approved it. Yet, occasionally, you will see that the FDA will ban certain products. Even with the products that the FDA says have been banned, you will still find them on the market. This do not make people take its integration seriously. So, the post-market surveillance should be more. It will put the TM practitioners on their toes and they will not just release anything on the market. Once there is sanity in the TM system, people will endorse the integration”.*

*[Participant 2, PM, Kumasi]*

In addition, some of the participants suggested that TM practitioners should state important health information such as expiry dates, dosage, storage of TM products, and therapeutic effects of TM products. They believed that the availability of such information would ensure users’ safety and improve the integration process. Medical doctors added that the provision of such information would make health practitioners aware of the diseases TM products can treat and they would accept its integration.

*“For every product, there is the grand ml, the manufacturing date, expiry date, and how you will take it, the storage and those things. Therefore, when these things are clearly labelled on TM containers, then it will ensure that the user do not go beyond or below the dosage and it will improve TM practice and its integration”.*

*[Participant 1, PM, Offinso north]*

*“…it is very critical to label TM with the expiry date. When practitioners do that, they boost the public’s confidence in taking TM products and it can fit well in the formal health system”.*

*[Participant 7, MD, Kumasi]*

*“We should know the specific ingredients a TM product contain and the specific therapeutic effect it can address rather than being all over the place. When therapeutic effect is known then we will know, the specific disease conditions it will treat and its integration will be welcomed”.*

*[Participant 4, MD, Kumasi]*

#### 3.3.2. Comprehensive Health Insurance

Participants recommended that government should expand the national health insurance to include TM products, and their justification for a more comprehensive health insurance scheme was to increase patronage of approved TM products. They further explicated that incorporating TM in education and research would expose health practitioners to the scope and dynamics in both traditional and orthodox health systems.

*“It is like the hospital; when a pregnant woman comes to the hospital, I will take her to the maternity unit, which is not the hospital. We have different wings but we are all working in concert to achieve a common goal for our clients. So, if you have two different systems, then merging them in all spheres, be it education, research and service delivery would be great. So, I think integrative approach is the best because both practitioners would be exposed to the two systems”.*

*[Participant 3, MD, Offinso north]*

*“TM inclusion in the NHIS will help! I told you that some of the TM are costly. I am referring to the approved ones at pharmacies and clinics. So, if the insurance can cater for that, then it will be good. It will enable people to patronise the approved TM products”.*

*[Participant 1, NS, Offinso north]*

#### 3.3.3. Training of TM 

One important suggestion made by the participants is that the government should include TM in the medical school curriculum. Participants believed that the inclusion of TM in medical school curriculum would help orthodox health practitioners to familiarise themselves with issues relating to the TM field. Closely related to TM inclusion in medical school curriculum is increase in TM departments. Hospital administrators indicated that it is necessary for policymakers to expand the institutional base of TM by creating TM departments in all universities in Ghana so that the number of TM departments or schools would increase. Moreover, some of the participants concluded that for integration to work effectively there is the need for TM practitioners to receive professional training. Professional training of TM practitioners was seen as a means to expand the human resource in the field of TM and lead to the availability of such practitioners in various health facilities.

*“For integration to work, then they need to include the TM in our medical school curriculum. That way, I can appreciate what that field of medicine is about and working with TM practitioners would not be a problem”.*

*[Participant 4, MD, Kumasi]*

*“It will be great to establish TM departments in all universities in Ghana. We have many people who want to go to the university so, if we increase the number of TM departments, we will get more professionals in the field of TM, and the system will be improved”.*

*[Participant 5, HA, Offinso north]*

*“There is also the need to increase the human resource. We need to train more people in TM so that we will have more qualified TM practitioners to practice in the various hospitals”.*

*[Participant 7, PM, Kumasi]*

#### 3.3.4. Improved Communication Strategy

Given that the nature of communication in the health system was not encouraging, participants recommended that the government must develop a communication strategy to improve sensitisation of the public about TM integration into the formal health system. They stated that both audio and audio-visual means of communication could be used for the circulation of the information. Participants believed that publicly explaining and promoting TM integration could make people understand the practice of integrated healthcare and make informed decisions by determining the kind of health service they want.

*“There should be more education through the media; especially radio and television to sensitise the people about the integration. So that people will come to understand that, there is an established health system where users can decide the type of healthcare they want”.*

*[Participant 1, HA, Kumasi]*

*“We need to increase publicity about the integration of the two health systems. There is no point for the integration if it only exist on paper. We can sensitise health users and practitioners about the system through community radio stations and even television stations”.*

*[Participant 2, NS, Offinso north]*

## 4. Discussion

This study explored the experiences and recommendations of orthodox health practitioners and hospital administrators on the practice of integrated healthcare in Ghana within the Kumasi metropolis and Offinso north district. One key contribution of the study to existing literature is the participants’ recommendation for improved TM integration in Ghana. The health governance, financing, and health architecture sections of the framework for integrating TM into national health systems [6] served as the theoretical basis for the study. 

Regarding the Ghanaian health governance and financing structure, study participants acknowledged the existence of TM regulatory bodies and Act but were not satisfied with the implementation of the policies governing TM practice in Ghana. This finding confirms the results of other Ghanaian studies [5,29,45] where inefficient implementation of TM policies and regulations were identified as some of the factors hindering the practice of integrated healthcare in Ghana. The ineffective implementation of TM policies as well as unsatisfactory performance of TM regulatory bodies could be an obstruction to TM integration into the Ghanaian health system. This is because well implemented regulations by specialist bodies are identified as a pre-condition for effectively integrating TM into national health systems [6].

It is reported that financial accessibility promotes equity in accessing healthcare [6]. The popular notion that TM is affordable [3,46] is debateable in this study. The current study has indicated that the cost of TM products and services is reliant on the nature of practice. Thus, TM products offered within formal health settings tend to be expensive, whereas community-based TM products were considered cheaper. This result is in line with the work of Ahlberg [47], who reported that some TM products are more expensive than orthodox healthcare, yet service users patronised them because such product/services tended to satisfy their healthcare needs [47]. The basic goal for integrating TM into formal health systems is to expand the scope and access to health services [2,6] among populations. The affordability of community-based TM products might promote the attainment of this goal; however, it could expose users to health complications due to safety issues. On the other hand, the expensive nature of approved TM products might thwart the success of TM integration since users would pay out of pocket. 

Directly related to financial accessibility of TM is the nature of NHIS coverage in Ghana. Participants noted that NHIS subscription is high with most subscribers being economically challenged. It was reported that the scheme covers only the orthodox health system. Clearly, TM integration was not considered in the key national healthcare financing scheme. In Ghana, the NHIS has positively influenced health service utilisation among the populace [28,48,49,50] because service users do not pay for medicines on the NHIS drug list. This evidently shows that the inclusion of TM products in national health cover remains a sure way of properly integrating TM into formal health systems [6]. China and Korea, with effective integrated health systems, have included TM in their national insurance cover, thereby leading to a reduction in out of pocket refund for TM products and services [6,51]. Possibly, the exclusion of TM products from the Ghanaian NHIS might have contributed to the inefficient state of the country’s integrated health system since service users have to pay for TM products out of pocket. 

A common concern raised by the study participants is the low level of professional or formal training among TM practitioners. Orthodox health practitioners and hospital administrators were concerned about TM practice because of the presumed inadequate professional training and knowledge among TM practitioners. Participants reported that most practitioners in the TM field acquired their knowledge informally from generation to generation. This observation is consistent with earlier studies in Africa [5,45,52,53] that orthodox health practitioners disregard TM practice due to the low level of medical education among its practitioners. The presumed inadequate level of professional or formal education among TM practitioners could be an obstacle to effective integration of TM into the Ghanaian health system. Well-trained TM practitioners as well as sound educational systems on TM are needed to develop a firm and efficient integrated system [6]. Therefore, the government of Ghana could expand TM training institutions to promote robustness in TM regulation and training.

It seems that the practice of inclusive health system in Ghana is more about paying lip service. Ideally, in an inclusive health system, TM regulation as well as training of TM practitioners should improve, while the cost of healthcare decreases. However, this is not the case in Ghana as participants recounted that TM products offered at formalised settings including integrated health facilities are expensive. The high cost of TM products at integrated health facilities and TM clinics and the exclusion of such products from the NHIS might continue to plague the Ghanaian health system. However, an appropriate health governance and financing structure could provide financial support for TM practice and aid proper training of TM practitioners to promote trust and respect between and among the health practitioners.

Reflecting on the Ghanaian health architecture, this study shows that knowledge about the practice of integrated healthcare is high among study participants. Participants, irrespective of professional background and location of affiliated institutions, were aware of the presence of integrated health facilities and the TM Department at KNUST. This finding confirms the result of a Ghanaian study [25] and a systematic review that assessed the effectiveness of integrated health systems in Africa [27]. The framework for integrating TM into national health systems clearly specifies that the availability and acquisition of health information is crucial to the building of an effective integrated system [6]. This means that the awareness of orthodox health practitioners and hospital administrators regarding the practice of integrated health could be a positive reinforcement to the implementation of the integration intervention.

Most participants perceived TM as the oldest health system. A similar finding has been reported by Tabi et al. [54], who indicated that TM is part of the Ghanaian culture. In addition, some of the participants deemed that TM use is associated with minimal side effects. This report has also been observed in other studies [55,56]. The perception that TM use presents the least form of adverse reactions was popular among hospital administrators. Acknowledgement of TM as the oldest form of healthcare in the Ghanaian setting coupled with its perceived minimal side effects could serve as enabling factors to TM integration, because notable and historic use of traditional therapies among a given population is a motivation to promote the integration process [6].

Similarly, participants also considered the availability of medicinal plants and ready market for TM as opportunities or prospects that could cause TM integration to flourish. They envisaged that the incorporation of TM has the potential of increasing patronage of health services and making the integrated health unit sustainable. This observation has also been noted in a study [57]. The availability of TM in the Ghanaian ecosystem serves as a facilitator to its integration into the formal health system because resource availability as well as communication are some of the key elements needed to build a functional integrated health system [6].

Communication about and within the integrated system was found to be poor. Study participants reported that service users do not willingly disclose their use of TM to orthodox health practitioners. The works of Agyei-Baffour et al. [24] and Peprah et al. [37] support this finding. Likewise, cross-referral is a vital means of assisting TM integration into national health systems [5]. However, our study demonstrates that the level of referral between the two health practitioners is minimal and mostly informal. This report is consistent with findings of studies conducted in Ghana, South Africa, Tanzania, and other sub-Saharan African countries [5,21,58,59,60]. One of the requirements necessary for proper integration is the acceptance of TM use and its integration by orthodox health practitioners [6]. Therefore, the stern opposition by orthodox health practitioners to refer service users to TM practitioners might obstruct the practice of an integrated healthcare in Ghana. The policy on TM practice in Ghana endorses the training of media experts to help educate the populace on the safe use of TM [61]. Therefore, to increase acceptability of TM among orthodox health practitioners, the media, particularly radio and television, could actively engage in publicising the integrated health system.

Study participants recounted that conducting medical examinations on service users and constant verification among orthodox health practitioners facilitate the delivery of quality healthcare, which is a classic characteristic of orthodox medicine [47]. It is worth mentioning that none of the integrated health studies conducted in the study area have reported such a finding, hence making this finding a unique contribution to the published literature. 

The inclusion of TM into the Ghanaian formal health system shows the determination of the government in expanding healthcare delivery in the country. This was evident in the findings of the study as all four groups of participants reported emphatically that TM integration has led to the availability or provision of options in health services. This astute finding has not been highlighted in the literature because most integrated health studies report on perceived rather than empirical benefits of integration [33,62]. Moreover, hospital administrators believed that the practice of integrated healthcare has helped the country by boosting its database on health accessibility and the provision of safe TM products and services. To the best of our knowledge, this finding has not been presented by any of the integrated health studies in the study setting. Besides these benefits, medical doctors felt that TM integration has led to the preservation of indigenous medicine. This finding is consistent with previous studies that the merger of orthodox and traditional health systems could make the modern health system culturally sensitive and preserve indigenous medical knowledge [33,63]. Clearly, the practice of integrated healthcare is noted for offering alternative healthcare to populations [6]. Therefore, the provision of options in health services associated with TM integration into the Ghanaian integrated health system could motivate policymakers and other stakeholders to continue to implement the intervention even in the face of numerous challenges.

Study participants disclosed numerous challenges hindering TM integration into the formal health system in Ghana. These challenges are multifactorial in nature. As some of the challenges are attached to TM practice, others affect the delivery of, and access to, integrated health services. Participants’ major concerns included the non-certification or documentation of TM products, standardisation, regulatory issues, level of professional training among TM practitioners, and the absence of a written protocol to spell out details about the integration. These concerns have been raised in earlier studies conducted in Ghana and other African countries [5,25,26,29,33,58,64]. Acceptance and involvement of health practitioners, particularly medical doctors, is a prerequisite for effective integration [6]. Therefore, opposition by medical doctors to TM usage could be a hindrance to the successful integration of TM into the Ghanaian health system. Until the above-mentioned concerns are addressed, the Ghanaian health system will continue to be non-functional and ineffective. 

To some extent, integrating TM into the Ghanaian health system has received positive reactions from stakeholders, with the perception that the consolidated health unit would enhance health delivery in Ghana [33]. However, poor health governance/financing policies have negatively affected certain aspects of the Ghanaian health architecture. Despite the shortfalls of the system, some level of integration or collaboration exist at piloted health facilities [25]. To improve the level of integration between the two health systems, inclusion of TM in the medical school curriculum, strict regulation of TM practice, and an improved communication strategy would be required.

### 4.1. Implications for Practice 

The findings of the study suggest that TM integration into the Ghanaian health system has not been effective due to weak referral system, inadequate training on TM, and non-fulfilment of vital conditions/standards needed to effectively integrate TM into the Ghanaian health system. Based on these findings, the following recommendations are made to the various stakeholders. The government should conduct recurring investigations/research among orthodox and TM practitioners to identify the desired integration approach to adopt and clearly specify the roles of the stakeholders. For example, the Ministry of Health should explore the views of the two health practitioners to determine whether TM integration should involve referral agreement with public health facilities. They should enable the establish indigenous health institutes where TM practitioners would work collectively or both health practitioners could execute their duties within the same hospital/clinic. The outcome of such investigations should be documented and shared with the health practitioners and other stakeholders. Implementing the desired integration approach could strengthen the referrals system between and among the health practitioners. Since the two health systems are founded on different philosophies, the government should encourage knowledge sharing among the health practitioners through professional/formal training. Knowledge sharing among the two health practitioners would intensify their understanding of the underlying philosophies of health from the perspectives of TM and orthodox forms of care. Finally, to increase patronage of integrated healthcare services, users must be aware of the formal incorporation of TM into the Ghanaian health system. The government must subsidise the cost of approved TM products. Once products are subsidised, people especially the poor can afford such products when the need arises. 

### 4.2. Strengths and Limitations

One major strength of the study is the presentation of the experiences of four different groups of stakeholders from the formal health setting within a single document. Thus, the nature of the Ghanaian integrated health system has been presented from both the practice and administrative perspectives. The availability of such multifaceted knowledge could be valuable to policymakers in modifying existing policies to improve the integrated system. The inclusion of participants from two contrasting geographical settings—Kumasi metropolis and Offinso north districts—strengthens the reliability of the study findings. However, the findings cannot be generalised due to the adoption of a qualitative research approach. More so, the study could be affected by overestimation or underestimation of the issues discussed since participants had to recollect their experiences. This limitation was addressed by checking the data with the participants.

## 5. Conclusions

This qualitative study has demonstrated that TM integration into the Ghanaian formal health system has led to the availability of options in health services. This benefit of the system could serve as a facilitator to the integration process. Although participants supported and noted the benefits of an integrated health system in Ghana, they identified a number of socio-economic and political factors such as poor processing and packaging of TM products, highly priced TM products, and opposition of medical doctors to TM use as factors impeding the integration. Other challenges include inadequate publicity about TM integration and the absence of a protocol guiding the integration process. Strategies such as proper implementation of regulatory rules, proper processing and certification of TM products, professional training of TM practitioners, inclusion of TM in medical school curriculum, and educating the public about the practice of integrated health system through the media were suggested by participants as ways to improve the Ghanaian health system. Future research should focus on assessing the opinions and involvements of TM practitioners regarding the integration of traditional therapies into national health systems because proper teamwork through knowledge sharing and efficient management of authority or power in the system between orthodox and TM practitioners is critical in strengthening health systems [21].

## Data Availability

The data presented in this study are available on request from authors.

## Supplementary Materials

The following are available online at https://www.mdpi.com/article/10.3390/ijerph182111200/s1, Table S1: COREQ Checklist.

## Abbreviations

COREQ	Consolidated Criteria for Reporting Qualitative Studies
FDA	Food and Drug Authority
GHC	Ghana Cedis
GHS	Ghana Health Service
IGF	Internally Generated Funds
KNUST	Kwame Nkrumah University of Science and Technology
MD	Medical Doctor
NHIS	National Health Insurance Scheme
NS	Nurse
OM	Orthodox Medicine
PM	Pharmacist
TM	Traditional Medicine
